# Cavitary pulmonary lesions following emerging lophomoniasis: A novel perspective

**DOI:** 10.1002/rcr2.908

**Published:** 2022-02-03

**Authors:** Amirmasoud Taheri, Mahdi Fakhar, Ali Sharifpour, Elham Sadat Banimostafavi

**Affiliations:** ^1^ Toxoplasmosis Research Center Communicable Diseases Institute, Iranian National Registry Center for Lophomoniasis (INRCL), Mazandaran University of Medical Sciences Sari Iran

**Keywords:** cavitary pulmonary lesions, chest CT scan, chronic cough, lophomoniasis

## Abstract

In this article, we report a patient referred to the clinic of Imam Khomeini Hospital in Sari, Mazandaran, northern Iran, with a 3‐month complaint of chronic cough and weight loss. According to the chest computed tomography scan, a cavity was detected in the upper lobe of the patient's left lung. The patient had no history of smoking or underlying diseases that predisposed him to cavitation, including tuberculosis. Then, bronchoscopy was performed for the patient, and bronchoalveolar lavage fluid was sent to the Iranian National Registry Center for lophomoniasis, and *Lophomonas blattarum* was detected in samples. Finally, the patient's symptoms were totally resolved by prescribing third‐daily metronidazole for 2 weeks.

## Graphical abstract

Cavitary lung lesions are caused by different types of diseases such as malignancies, infectious lung diseases, rheumatologic disorders and so on. Infectious diseases cause the majority of them. Bacteria, fungi and protozoa are the most critical pathogens. Among protozoa, there have been no reports that *Lophomonas* causes cavitation in the lungs. Ours is the first reported case in which lophomoniasis presented with cavities in a subject who had no underlying disease, never smoked, was without lung infection or even had no chronic pulmonary symptoms.

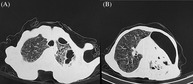

## INTRODUCTION

A variety of diseases, including cancer, rheumatologic and infectious lung diseases, can cause cavitary lung lesions.^1^ Among them, infections played the main role through their aetiological agents, such as bacteria, fungi and protozoa.^2^ Lophomoniasis involves the upper and lower respiratory tracts and causes fever, chronic cough, sputum production and so on. The disease is diagnosed by bronchoalveolar lavage fluid (BALF) obtained during bronchoscopy under a light microscope or by polymerase chain reaction (PCR) technique.^3^, ^4^ *Lophomonas blattarum* (L. blattarum) is a parasite that is responsible for the disease. It is a pear‐shaped protozoan that resides in the guts of cockroaches and helps them digest their food. These insects contaminate the human environment by excreting *Lophomonas* in their stools, and humans become infected by breathing the cyst‐containing aerosol.^4^, ^5^, ^6^ There are some evidence that report lophomoniasis bronchoscopy views in the infected patients.^5^, ^6^, ^7^ Here, we describe a patient who revealed cavitary lung lesions due to *Lophomonas* as an emerging and mysterious protozoan parasite.

## CASE REPORT

In June 2019, a 57‐year‐old male was referred to the Imam Khomeini Hospital clinic, Sari, Mazandaran province, northern Iran, with a 3‐month complaint of chronic cough and weight loss. During this period, he was on various medications, including pantoprazole tablets and Pulmicort Turbuhaler. However, the patient's symptoms did not improve. He has no underlying medical conditions and does not smoke or abuse drugs. The physical examination was also normal.

**FIGURE 1:**
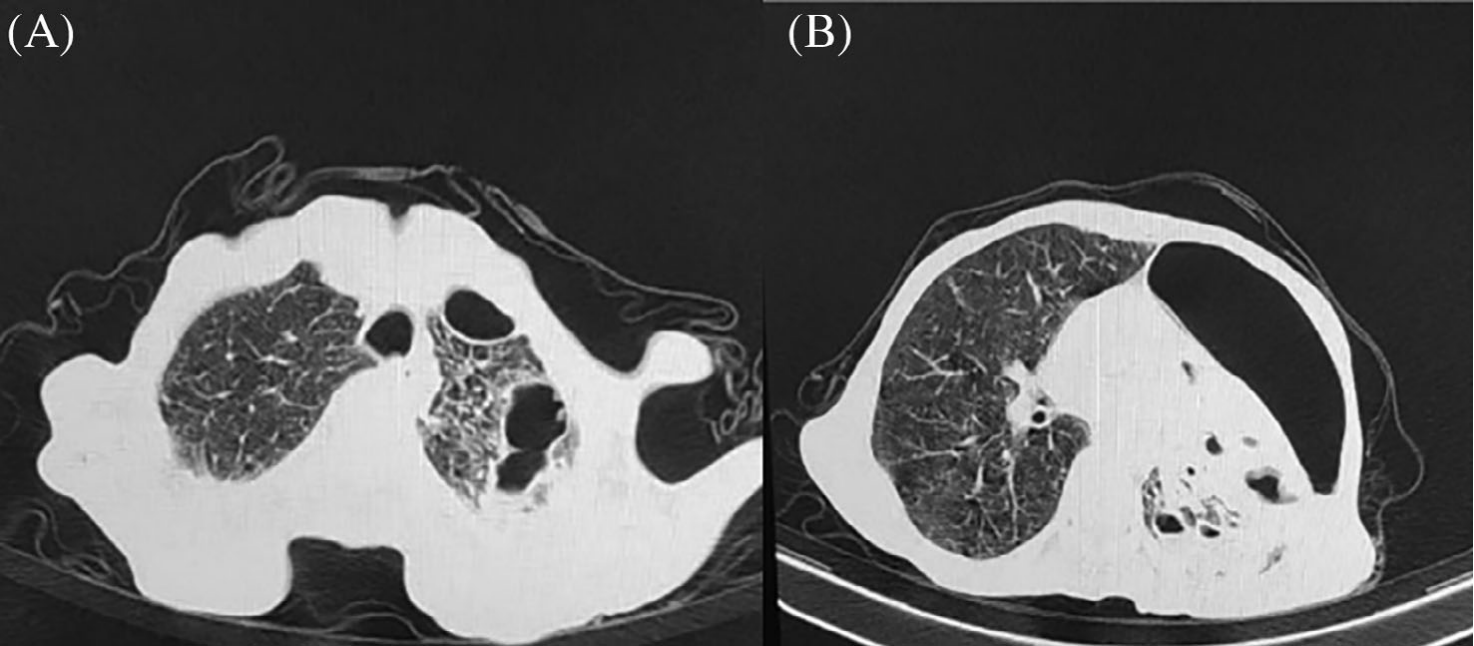
(A) Cavities in the left upper lobe (an axial section of the chest computed tomography [CT] scan) and (B) pneumothorax in the left upper lobe (an axial section of the chest CT scan)

A chest computed tomography (CT) scan demonstrated a cavitary lesion and pneumothorax in the left upper lobe of the lung (see Figure 1). The patient has no previous history of lung disease, such as tuberculosis, and the purified protein derivative (PPD) test resulted negative. A serum sample of the patient resulted negative in the mycological laboratory for the diagnosis of invasive pulmonary aspergillosis using galactomannan (GM) enzyme immunoassay (EIA) test, as an antigen‐based assay. Our last differential diagnosis was lung abscess; however, this diagnosis was also incompatible with the patient's clinical symptoms and history.

Next, he underwent a bronchoscopy, and BALF samples were then examined in the laboratory. Under a light microscope*, L. blattarum* with its moving flagella was detected. Nevertheless, the PCR technique was also done and endorses the light microscopy results. According to our experience in the Iranian National Registry Center for Lophomoniasis (INRCL),^8^ parasite severity index which the mild density was defined as 1–10 parasites/100 HPF, moderate as 1–10 parasites/10 HPF and 1–10 parasites/HPF were considered as severe, the patient had a severe type of lophomoniasis. For 2 weeks, he was given metronidazole tablets three times a day. At the 8‐month follow‐up visit, the patient's symptoms completely disappeared but he refused to get a chest x‐ray or a chest CT scan despite our requests.

## DISCUSSION

Cavitary lung lesions are caused by different types of diseases such as malignancies, infectious lung diseases, rheumatologic disorders and so on.^1^ Infectious diseases cause the majority of them. Bacteria, fungi and protozoa are the most critical pathogens.^2^ Among protozoa, there have been no reports that *Lophomonas* causes cavitation in the lungs. Ours is the first reported case in which lophomoniasis presented with cavities in a subject who had no underlying disease, never smoked, was without lung infection or even had no chronic pulmonary symptoms. Even though several studies report varied bronchoscopic images of lophomoniasis,^4^ none of them mention a lung cavity. This case should raise concerns about paying more attention to this recently emerged pulmonary disease. We conclude that in each patient with cavitary lung lesions, pulmonary lophomoniasis should be considered in the differential diagnosis by pulmonologists.

## CONFLICT OF INTEREST

None declared.

## AUTHOR CONTRIBUTION

Amirmasoud Taheri and Mahdi Fakhar wrote the manuscript. All authors contributed to editing of the manuscript and approved the final version of the manuscript.

## ETHICS STATEMENT

The authors declare that appropriate written informed consent was obtained for the publication of this manuscript and accompanying images.

## Data Availability

The data are available with the corresponding author and can be achieved on request.
